# Acute arterial stiffness responses to on-ball balance exercises in young and middle-aged adults: Role of posture and cumulative effects

**DOI:** 10.3389/fphys.2023.1081668

**Published:** 2023-02-17

**Authors:** Wei Chen, Gaofan Miao, Yanfei Xv, Zhixiong Zhou, Weili Zhu

**Affiliations:** Cardiovascular Health Laboratory, Capital University of Physical Education and Sports, Beijing, China

**Keywords:** swiss ball, balance exercise, arterial stiffness, postures, kneeling, sitting, cumulative effects

## Abstract

**Objective:** To examine the acute arterial stiffness changes after maintaining one bout of balance on Swiss ball using different postures in young and middle-aged adults, and to evaluate the cumulative exposure effects on arterial stiffness after multiple exercise bouts in middle-aged adults.

**Methods:** Using crossover design, we first enrolled 22 young adults (24.0 ± 1.1 years) and randomized them to non-exercise control (CON), on-ball balance exercise trial lasting 1 × 5 min in kneeling posture (K1) and sitting posture (S1). In a following crossover experiment, 19 middle-aged adults (53.0 ± 4.7 years) were randomized to non-exercise control (CON), on-ball balance exercise trial lasting 1 × 5 min in kneeling posture (K1) and in sitting posture (S1), and on-ball balance exercise trial lasting 2 × 5 min in kneeling posture (K2) and in sitting posture (S2). Cardio-ankle vascular index (CAVI), an indicator of systemic arterial stiffness, was measured at baseline (BL), immediately after (0 min), and every 10 min after exercise. CAVI changes from BL in the same trial (⊿CAVI) were used for analysis.

**Results:** In K1 trial, ⊿CAVI decreased significantly at 0 min (*p* < 0.05) in both young and middle-aged adults; however in S1 trial, ⊿CAVI at 0 min increased significantly in young adults (*p* < 0.05), with ⊿CAVI tending to increase in middle-aged adults. Bonferroni post-test revealed that at 0 min, ⊿CAVI of K1 in both young and middle-aged adults, and ⊿CAVI of S1 in young adults differed significantly from that of CON (*p* < 0.05). In middle-aged adults, ⊿CAVI decreased significantly at 10 min compared to BL in K2 trial (*p* < 0.05), and increased at 0 min compared to BL in S2 trial (*p* < 0.05); however, difference compared to CON was not significant.

**Conclusion:** Single on-ball balance bout in kneeling posture improved arterial stiffness transiently in both young and middle-aged adults; however, sitting posture elicited opposite changes, and this happened only in young adults. Multiple balance bouts resulted in no significant change in arterial stiffness in middle-aged adults.

## Introduction

Swiss ball is widely practiced in the fitness industry, and evidence shows that it is a good overall training tool for developing core strength, flexibility, and balance ability (Sekendiz et al., 2010; Young et al., 2015; Yu et al., 2017). Recently, ball exercise has been expanded to arterial stiffness regulation, and intervention studies reported that regular ball training increased body flexibility and simultaneously reduced arterial stiffness in middle-aged males (Ikebe et al., 2022a), and even acute trunk stretching using Swiss ball reduced arterial stiffness (Ikebe et al., 2022b). Therefore, it is believed that stretching component of ball exercises was responsible for arterial stiffness improvement (Ikebe et al., 2022a; Ikebe et al., 2022b). However, besides stretching exercises, often included in Swiss ball training protocols were on-ball balance exercises, for example, balancing on a ball in kneeling and sitting posture (Ikebe et al., 2022a). Obviously, these two exercises are associated with balance ability rather than flexibility. Since better balance ability was associated with decreased arterial stiffness (Peultier-Celli et al., 2021), it is likely that on-ball balance exercises *per se* might regulate arterial stiffness in humans.

When people balance on a Swiss ball, kneeling and sitting are the most often postures assumed (Ikebe et al., 2022a). To maintain different posture, different muscle groups are involved, probably at different contraction intensities. Based on the fact that different muscles (Okamoto et al., 2009; Li et al., 2015) and different intensities (Okamoto et al., 2013; Menêses et al., 2015) may determine arterial stiffness response, it is possible that on-ball balance exercise using different postures might result in distinct profile of arterial stiffness changes.

The effects of exercise can vary greatly among individuals. Specifically, physiological changes associated with aging may impact cardiovascular responses to exercise. It is also of note that the response depends on factors such as mode, intensity and duration of exercise (Liu et al., 2022). To optimize exercise prescription, and to make physical activity part of personalized medicine, it is fundamental to know whether health outcomes in young and middle-aged population respond in a consistent manner.

There is usually a dose-response relationship between physical activity and health benefit (Garber et al., 2011). However, greater benefit is not always associated with higher amounts of physical activity, even within a limited exercise volume range (Zhou et al., 2022). In terms of on-ball balance exercises, it is of interest to uncover whether cumulative effect exists when additional sessions are performed.

Therefore, this study aimed to examine the immediate effects of one bout of on-ball balance exercise using different postures on arterial stiffness in healthy young and middle-aged adults. The secondary purpose was to examine whether the arterial stiffness changes could be enhanced by additional sessions in middle-aged adults.

## Materials and methods

Twenty-two healthy young adults (22 females aged 24.0 ± 1.1 years) and 19 middle-aged adults (15 females and 4 males aged 53.0 ± 4.7 years) participated in the study. All the subjects enrolled had never practiced ball exercise prior to this study. Written informed consents were obtained before the data collection. The study was approved by the Ethics Committee of Capital University of Physical Education and Sports (2022A04), and the protocol was registered through Chinese Clinical Trial Registry (ChiCTR2200055940).

### Design

**FIGURE 1:**
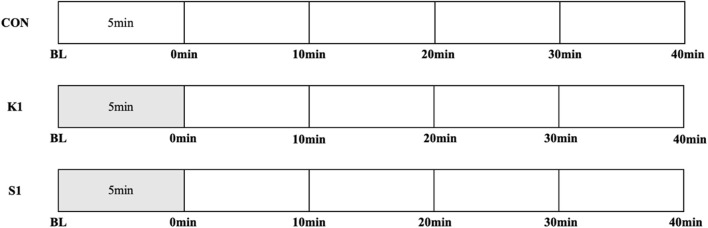
Study protocols for exercise, recovery, and measurements in three trials in young adults: non-exercise control trial (CON), on-ball balance exercise in kneeling posture and sitting posture for 1 × 5 min (K1 and S1, respectively). Time points of measurements were baseline (BL), immediately (0 min), 10 min, 20 min, 30 min, and 40 min after exercise.

**FIGURE 2:**
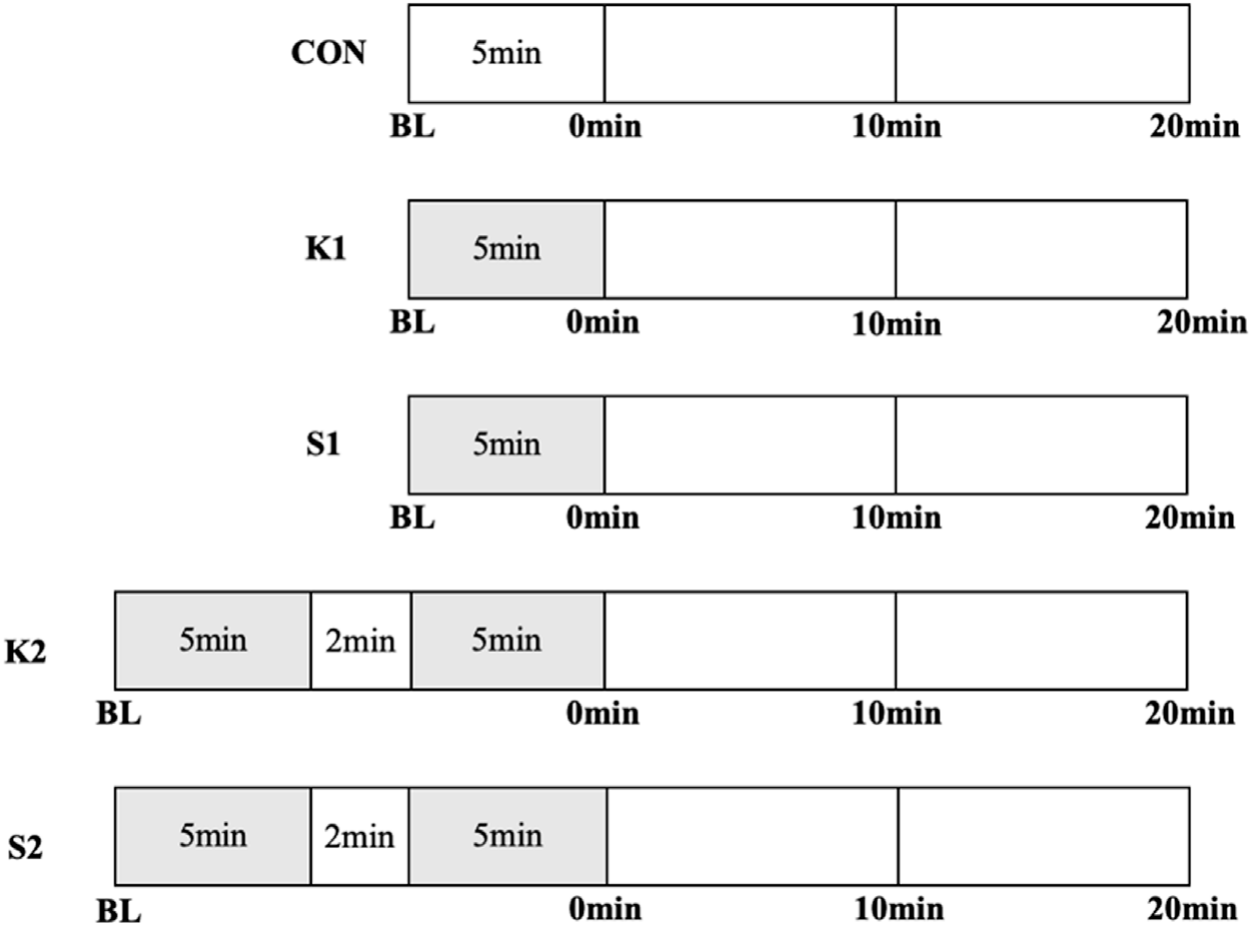
Study protocols for exercise, recovery, and measurements in five trials in middle-aged adults: non-exercise control trial (CON), 1 × 5 min and 2 × 5 min on-ball balance exercises in kneeling posture (K1 and K2, respectively) and in sitting posture (S1 and S2, respectively). Time points of measurements were baseline (BL), immediately (0 min), 10 min, and 20 min after exercise.

Young adults took part in three trials in random order: non-intervention control (CON), 1 × 5 min on-ball balance exercises in kneeling posture (K1) and in sitting posture (S1), as seen in Figure 1. Middle-aged adults participated in five trials in random order: non-intervention control (CON), 1 × 5 min and 2 × 5 min on-ball balance exercises in kneeling posture (K1 and K2, respectively) and in sitting posture (S1 and S2, respectively), as seen in Figure 2. All subjects underwent only one trial during one visit to the lab, and successive visits were separated by 1 week. It took three visits across 3 weeks for young adults, and five visits across 5 weeks for middle-aged adults, to complete the study.

Measurements were performed at six time points in each trial of young adults, and four time points in each trial of middle-aged adults. In CON, subject underwent no intervention except measurements, and was in supine position quietly throughout the trial. In K1, S1, K2 and S2 trials, subject maintained balance on the Swiss ball in corresponding posture after baseline measurement.

Subjects were asked to maintain their physical activity habits throughout the study period, except undergoing ball exercise protocol according to our design. Prior to each trial, subjects were instructed to refrain from alcohol, caffeine and strenuous physical activity for 48 h. Subjects visited the lab after a light meal, and were at least 3 h post-prandial in the afternoon. They rested in sitting position for 15 min, and then in supine position for 2 min before baseline measurement. During the period of 2-min supine position, electrodes, microphone, and cuffs were applied to the subjects. Room temperature ranged from 22°C to 25°C.

### On-ball balance exercises

**FIGURE 3:**
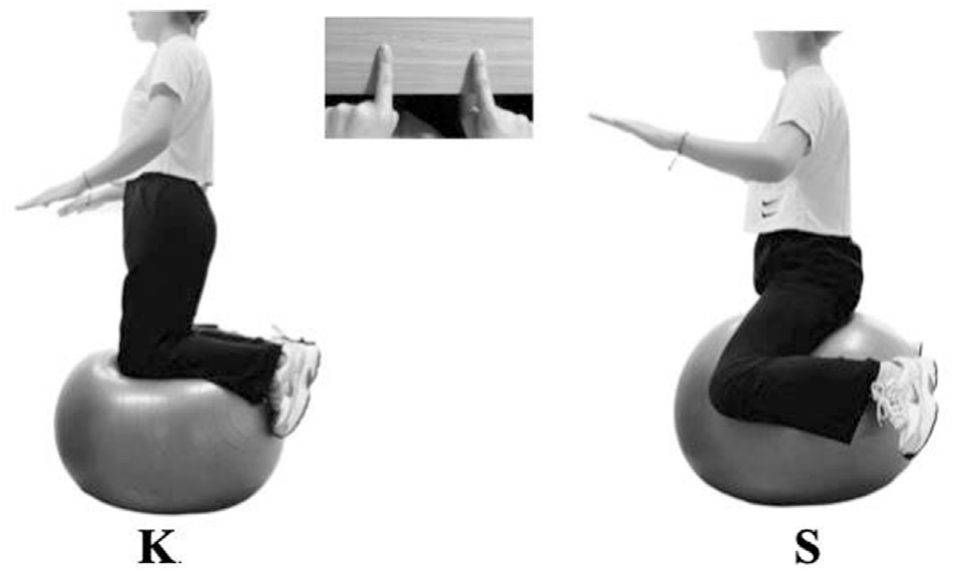
On-ball balance exercise in kneeling and sitting posture. K: on-ball balance exercise in kneeling posture; S: on-ball balance exercise in sitting posture. K and S were equal to exercise II⑥ and I④, respectively, in Figure 1 from Ikebe et al. (2022a).

Subjects were asked to maintain balance on a Swiss ball (JOINFIT, FYJ007), 75 cm in diameter, in kneeling and sitting posture. In the former posture, subjects were asked to kneel and balance on the ball, with knees flexed approximately 90° and trunk erected. The sitting posture was performed with buttocks on the ball, legs straddling the ball, and feet off the ground, with knees squeezing the ball to maintain balance (Figure 3). During the exercises, when the subjects were to lose balance and fall off the ball, they were allowed to touch a stable table in front of them by their fingertips, so to get the lightest support needed to continue the balance. The examiner stood closely behind the participant to give the lightest touch, when necessary, to prevent participant’s falls and fall injuries.

### Measurements

Arterial stiffness in cardio-ankle vascular index (CAVI) was measured using a VaSera VS-1500 vascular screening system (Fukuda Denshi, Beijing, China). With the subjects in supine position, electrocardiogram electrodes were placed on both wrists, a microphone for monitoring heart sounds (phonocardiogram) was placed on the sternum, and four cuffs were wrapped around the upper arms and ankles as instructed. When the electrocardiogram was stable and the first and second heart sounds were detected in the phonocardiogram, the START button was pressed, and the values of right and left CAVI were obtained using the system completely independent of human operation. Cuffs and electrodes were left on the participants so to measure CAVI at different time points efficiently, and were removed only during the balance exercise period. At each time point, one measurement was performed for record.

Measurements of blood pressure and heart rate were performed simultaneously by the vascular screening system.

### Statistical analysis

Statistical processing was performed using GraphPad Prism Software 9.0. All data are expressed as mean ± SD. Mauchly’s test of sphericity was performed to examine whether the assumption of sphericity was met. The responses of ⊿CAVI, blood pressure and heart rate to intervention were analyzed by two-way (treatment × time) ANOVA of repeated measures, with Bonferroni post-test to determine the time point at which the difference across trials occurred. Sample size was estimated using G*Power (Version 3.1). Based on a *ß* error of 20% (power = 0.80), an α error of 0.05, and effect size of 0.25 (Ikebe et al., 2022a; Ikebe et al., 2022b), a total sample size of 36 for young adults (12 sample size per trial), and 50 for middle-aged adults (10 sample size per trial), was suggested separately. Statistical significance level was set at *p* < 0.05.

## Results

Subjects’ characteristics are presented in Table 1. Age, weight and body mass index (BMI) were significantly different between the young and middle-aged groups. Middle-aged adults had higher blood pressure than young adults (*p* < 0.01). As expected, middle-aged adults also had higher CAVI (*p* < 0.01) compared with young group, which indicated that the arteries of middle-aged adults were stiffer.

**TABLE 1:** Subject characteristics.

	Young (*N* = 22)	Middle-aged (*N* = 19)	*p*
Age (years)	24.0 ± 1.1	53.0 ± 4.7	<0.001
Height (cm)	163.5 ± 5.6	161.3 ± 7.2	0.281
Weight (kg)	53.2 ± 6.6	67.9 ± 8.8	<0.001
Body mass index (kg/m^2^)	19.9 ± 2.0	26.1 ± 3.0	<0.001
Systolic BP (mmHg)	110 ± 9	126 ± 18	0.001
Diastolic BP (mmHg)	67 ± 7	81 ± 11	<0.001
CAVI	6.0 ± 0.8	7.6 ± 0.7	<0.001

Values are mean ± SD.

BP: blood pressure, CAVI: cardio-ankle vascular index.

The blood pressure here was measured at baseline in CON trial.

**FIGURE 4:**
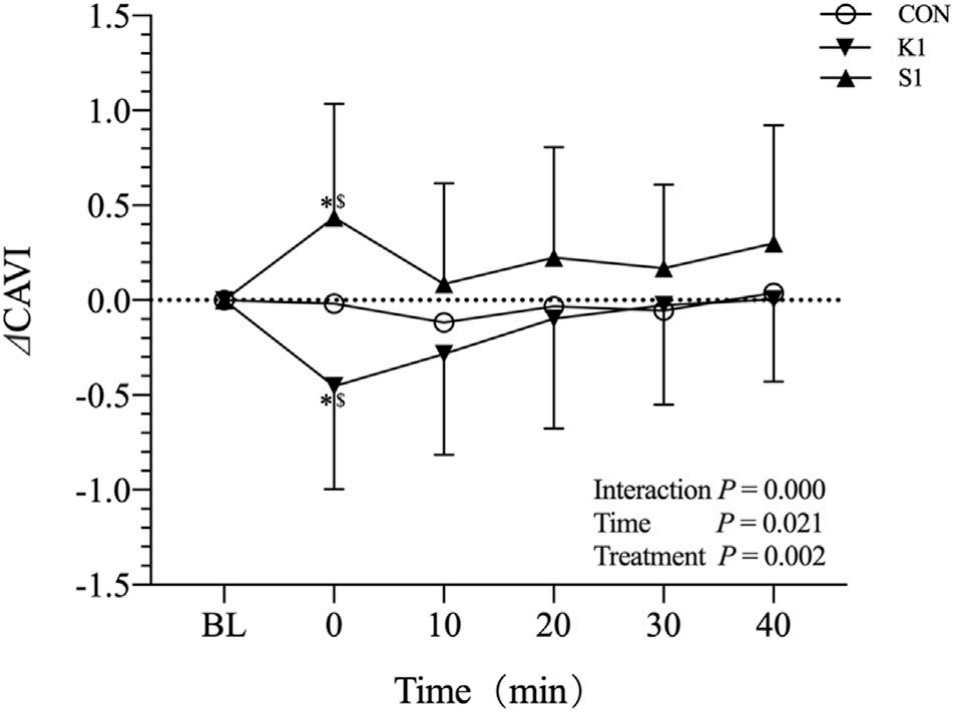
Mean (±SD) time-dependent ⊿CAVI changes undergoing CON, K1, and S1 trials in young adults. Statistical analysis was performed using two-factor (treatment and time) ANOVA with repeated measures with Bonferroni post-tests. Data are mean ± SD, *n* = 22. ^*^ *p* < 0.05: 0 min vs. BL in K1 and S1 trial. ^$^ *p* < 0.05: K1 and S1 vs. CON at 0 min. CAVI, Cardio-ankle vascular index; BL, baseline; CON, control; K1: on-ball balance exercise in kneeling posture for 5 min, S1: on-ball balance exercise in sitting posture for 5 min.

The responses of ⊿CAVI to CON, K1 and K2 in young adults are shown in Figure 4. There was no significant ⊿CAVI changes with time in CON trial. However, ⊿CAVI decreased to −0.45 ± 0.53, and increased to 0.44 ± 0.59, at 0 min in K1 and S1 trial, separately. Bonferroni post-test revealed that, at the time point of 0 min, ⊿CAVI in K1 trial was significantly lower than that in CON (*p* < 0.05), and ⊿CAVI in S1 trial was significantly higher than that in CON (*p* < 0.05).

**FIGURE 5:**
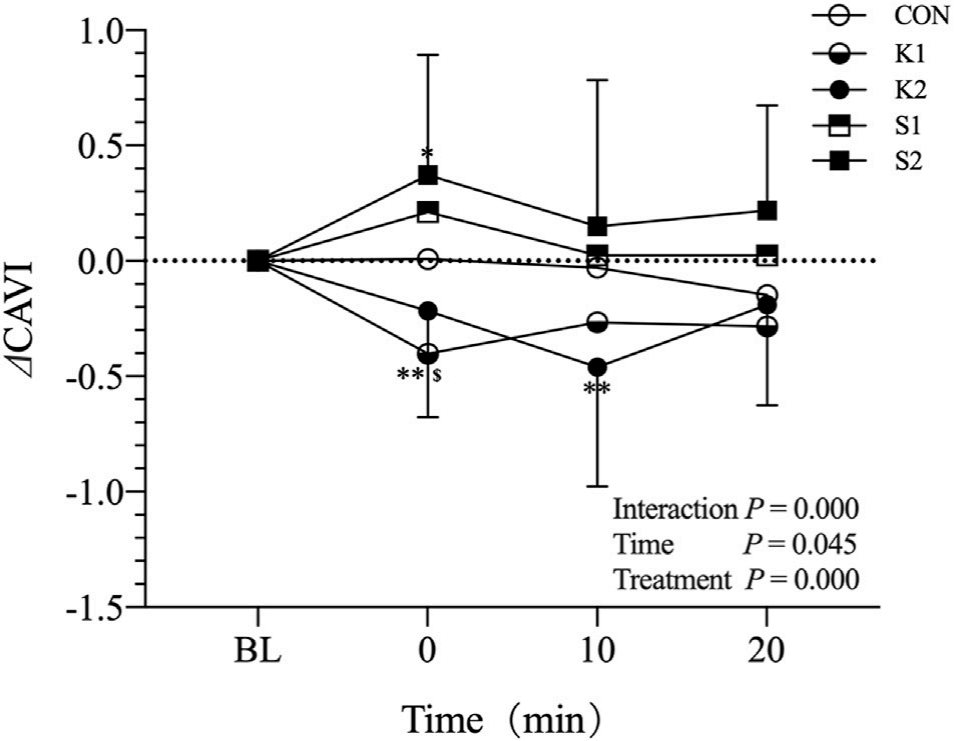
Mean (±SD) time-dependent ⊿CAVI changes undergoing CON, K1, K2, S1 and S2 trials in middle-aged adults. Statistical analysis was performed using two-factor (treatment and time) ANOVA with repeated measures with Bonferroni post-tests. Data are means ± SD, *n* = 19. ^**^ *p* < 0.01, 0 min vs. BL in K1 trial, 10 min vs. BL in K2 trial. ^*^ *p* < 0.05, 0 min vs. BL in S2 trial, ^$^ *p* < 0.05, K1 vs. CON at 0 min. CAVI, Cardio-ankle vascular index; BL, baseline; CON, control; K1: on-ball balance exercise in kneeling posture for 5 min, S1: on-ball balance exercise in sitting posture for 5 min, K2: on-ball balance exercise in kneeling posture for 2 × 5 min, with 2 min rest in between, S2: on-ball balance exercise in kneeling posture for 2 × 5 min, with 2 min rest in between.

Figure 5 shows the changes of ⊿CAVI in CON, K1, S1, K2 and S2 trials in middle-aged adults. ⊿CAVI remained unaltered with time in CON and S1 trial. In K1 trial, ⊿CAVI decreased significantly with time (0.00 ± 0.00, −0.40 ± 0.43, −0.27 ± 0.46 and −0.28 ± 0.60 at BL, 0 min, 10 min, and 20 min, respectively). ⊿CAVI decreased significantly to −0.46 ± 0.50 at 10 min in K2 trial, however, increased significantly to 0.37 ± 0.51 at 0 min in S2 trial, relative to their corresponding BL. Bonferroni post-test revealed that, at the time point of 0 min, ⊿CAVI in K1 trial was significantly lower than that in CON (*p* < 0.05). However, the ⊿CAVI differences with time in K2 and S2 trial were not significant relative to CON condition.

As indicated in Table 2, systolic blood pressure increased immediately after kneeling exercise in young adults compared with the CON (*p* < 0.01). Blood pressure did not demonstrate significant time-dependent changes in middle-aged adults in response to the balance exercises, as shown in Table 3.

**TABLE 2:** Effects of on-ball balance exercises on blood pressure in young adults (*N* = 22).

	BL	0 min	10 min	20 min	30 min	40 min	*p* value
Heart rate (beats/min)							
CON	65 ± 7	66 ± 7	64 ± 6	64 ± 7	64 ± 7	65 ± 8	T = 0.000
K1	68 ± 6	73 ± 8	69 ± 7	67 ± 8	68 ± 8	67 ± 9	G = 0.137
S1	67 ± 6	69 ± 7	66 ± 6	66 ± 8	64 ± 6	65 ± 7	I = 0.301
Systolic blood pressure (mmHg)							
CON	110 ± 9	109 ± 11	107 ± 12	108 ± 10	107 ± 11	107 ± 8^*^	T = 0.000
K1	110 ± 11	116 ± 12**,^†^	109 ± 11	108 ± 11	109 ± 10	110 ± 11	G = 0.706
S1	109 ± 8	110 ± 8	108 ± 10	107 ± 11	107 ± 10	107 ± 8	I = 0.002
Diastolic blood pressure (mmHg)							
CON	67 ± 7	66 ± 7	66 ± 6	66 ± 5	66 ± 5	67 ± 7	T = 0.001
K1	67 ± 6	69 ± 6	67 ± 8	65 ± 7	66 ± 7	66 ± 7	G = 0.893
S1	67 ± 6	67 ± 6	66 ± 7	65 ± 6	66 ± 7	66 ± 7	I = 0.518

Values are mean ± SD.

BL, baseline; CON, control; K1, on-ball balance exercise in kneeling posture for 1 × 5 min; S1, on-ball balance exercise in sitting posture 1 × 5 min; T, time; G, group; I, interaction.

**p < 0.01 vs. BL.

^†^ *p* < 0.05 vs. CON.

**TABLE 3:** Effects of on-ball balance exercises on blood pressure in middle-aged adults (*N* = 19).

	BL	0 min	10 min	20 min	*p* value
Heart rate (beats/min)					
CON	70 ± 5	69 ± 4	68 ± 5	69 ± 6	T = 0.002
K1	70 ± 6	71 ± 8	69 ± 5	70 ± 6	G = 0.137
S1	69 ± 6	73 ± 6	71 ± 6	70 ± 7	I = 0.457
K2	70 ± 4	74 ± 6	70 ± 6	72 ± 4	
S2	70 ± 4	71 ± 5	67 ± 4	69 ± 6	
Systolic blood pressure (mmHg)					
CON	126 ± 18	127 ± 19	126 ± 17	126 ± 16	T = 0.000
K1	129 ± 18	132 ± 19	126 ± 16	125 ± 15	G = 0.987
S1	125 ± 17	128 ± 19	126 ± 17	124 ± 19	I = 0.290
K2	127 ± 20	130 ± 17	125 ± 17	125 ± 16	
S2	131 ± 19	130 ± 18	128 ± 19	125 ± 18	
Diastolic blood pressure (mmHg)					
CON	81 ± 11	83 ± 9	81 ± 10	81 ± 11	T = 0.008
K1	82 ± 15	83 ± 11	81 ± 11	81 ± 10	G = 0.983
S1	79 ± 11	81 ± 11	80 ± 11	80 ± 12	I = 0.980
K2	82 ± 14	82 ± 14	81 ± 13	80 ± 12	
S2	80 ± 13	83 ± 12	82 ± 13	81 ± 13	

Values are mean ± SD.

BL, baseline; CON, control; K1, on-ball balance exercise in kneeling posture for 1 × 5 min; S1, on-ball balance exercise in sitting posture for 1 × 5 min; K2, on-ball balance exercise in kneeling posture for 2 × 5 min, with 2 min rest in between; S2, on-ball balance exercise in sitting posture for 2 × 5 min, with 2 min rest in between; T, time; G, group; I, interaction.

## Discussion

Our principal finding is that, when the exercises were examined separately, on-ball balance exercises did not necessarily exert beneficial effect on arterial stiffness, with the posture being a critical determinant. Specifically, one bout of balance exercise in kneeling posture transiently decreased arterial stiffness in both young and middle-aged adults. Sitting posture, however, increased arterial stiffness in healthy young adults, and had not impact in middle-aged adults. Interestingly and surprisingly, when two bouts were performed in middle-aged adults, both the kneeling and sitting posture have no effect on arterial stiffness relative to control.

### Kneeling posture improved arterial stiffness transiently in young and middle-aged adults

Kneeling is a posture employed in a variety of environments and situations (Mezzarane and Kohn, 2008), and training on the knees has been used in physical therapy to improve postural control (Kurayama et al., 2013). In Swiss ball exercise, maintaining balance on the ball in kneeling posture is widely practiced. However, to the best of our knowledge, this has been used by only one study to modulate arterial stiffness, with the protocol being included in a combo of ball exercises (Ikebe et al., 2022a).

This study aimed to isolate the effects of on-ball balance from those of ball exercise combo used by Ikebe, H et al. (Ikebe et al., 2022a). Eliminating the confounding ball exercises, we asked the subjects to maintain balance on Swiss ball in kneeling position for 5 min, and the results showed that arterial stiffness decreased immediately after the exercise, and reverted gradually to baseline afterwards in both young and middle-aged adults. Since kneeling posture could result in calf ischemia, probably through popliteal artery kinking (Ramondou et al., 2021), and after the kneeling, the limb underwent reperfusion, thus constituting conditioning which might modulate arterial stiffness in humans (Kuusik et al., 2019), so it is likely that the transient improvement was associated with limb ischemia induced by kneeling.

### Unfavorable arterial stiffness changes induced by sitting posture in young adults

To combat sedentary behavior, sitting on Swiss ball rather than standard chair, usually termed as active sitting, is very popular in work settings. Active sitting is viewed as a simple way to increase daily physical activity while seated, and has been proved to increase energy expenditure (Beers et al., 2008; Dickin et al., 2017; Synnott et al., 2017) and muscle activation (Dickin et al., 2017). Though it seems that active sitting could benefit human health, no study has ever examined its effects on arterial stiffness.

In the sitting posture trial of this study, subjects were asked to sit on a ball with feet held off, rather than on the ground during active sitting (Dickin et al., 2017; Snarr et al., 2019; Buchman-Pearle et al., 2022). To our surprise, the results demonstrated that sitting posture increased arterial stiffness in young adults transiently, seemingly contradicting with the results of that long-term ball training improved arterial stiffness (Ikebe et al., 2022a). However, the improvement reported by Ikebe, H., et al. was induced by a ball exercise combo, rather than on-ball balance exercise in sitting posture *per se* (Ikebe et al., 2022a). So it was very likely that the unfavorable effect induced by sitting posture on arterial stiffness was neutralized, and even reversed by other exercises included in the combo.

Though regular aerobic exercise has been well documented to improve arterial stiffness, resistance training does not necessarily reduce arterial stiffness in humans (Miyachi, 2013), with acute dynamic resistance exercise elicited transient increase in arterial stiffness (DeVan et al., 2005; Yoon et al., 2010). However, the unfavorable arterial stiffness changes induced by balance exercise in sitting posture might be related to the subjects’ squeezing the ball with their knees, during which static contractions of leg muscles were obvious.

### No cumulative arterial stiffness changes induced by multiple bouts in middle-aged adults

Arterial stiffening is prominent with aging, and exercise strategies that improve arterial stiffening associated with aging are of importance to prevent cardiovascular disease. Usually, dose-response relationship exists between physical activity and health benefit (Haskell et al., 2007), however, it is not clear as to the specific quantity and quality of physical activity for the attainment of health benefits (Garber et al., 2011). Since this is the first study revealing the separate impact of on-ball balance exercise on arterial stiffness, it is of interest to further examine whether arterial stiffness benefits would accumulate when multiple balance bouts are performed.

In this study, two bouts of on-ball balance exercise were administered to examine cumulative exposure effects of on arterial stiffness in middle-aged adults. An interval of 2 min was used for the recovery, because a relatively short interval is necessary for cumulative improvement of arterial stiffness (Zheng et al., 2015). Though the decrease of arterial stiffness induced by kneeling and increase of arterial stiffness induced by sitting reached significance with time, the significant difference disappeared when compared with control condition. This supports our previous findings that increased amount of exercise did not inevitably elicit more benefit in arterial stiffness (Zhou et al., 2022). In the case of kneeling posture, the second bout of on-ball balance exercise even attenuated the impact of the previous bout. This is very similar to the results that a third bout of one-leg standing attenuating the previous two bouts (Zhou et al., 2022). So we proposed that the arterial stiffness responses to on-ball balance exercise and one-leg standing bear the most common quantitative feature of the hormetic-biphasic dose response, characterized by a low dose stimulation and a high dose inhibition (Calabrese, 2013). This has practical implication for exercise prescription aiming to improve cardiovascular health in humans.

## Limitations

The current study has limitations. First, it is hard to describe intensity of on-ball balance exercise, and hence impossible to set individualized intensity for each subject. However, Swiss ball exercise is a non-traditional type of exercise, and its intensity is an area that requires comprehensive work (Farlie et al., 2013). Another limitation is that, for purpose of accomplishing the task, middle-aged adults were more likely to rely on the fingertip touch to maintain balance, constituting the effect of light touch (Ishigaki et al., 2016). This effect may alter the exercise of muscles being used, and hence account for the absence of blood pressure response. However, middle-aged adults were encouraged to use the slightest touch to insure exercise intensity, and arterial stiffness changes occurred. Third, the muscle activity was not monitored. Future studies should examine electromyographical patterns of postural musculature, so to reveal possible mechanism of the distinct profiles of arterial stiffness changes. Finally, this is an acute intervention study, and the changes induced by balance exercise were transient. However, some of the major health-related changes produced by physical activity may be due more to “acute” biological responses during and for some time following each bout of activity than to a “training response” (Haskell, 1994). What’s more, there may be an interaction between these two types of responses (Haskell, 1994). Based on the fact that both acute ball exercise (Ikebe et al., 2022b) and training (Ikebe et al., 2022a) could improve arterial stiffness, the training effects of on-ball balance exercises deserve investigation.

## Summary

The main findings were that posture during on-ball balance exercise played critical role in the transient regulation of arterial stiffness, with kneeling posture decreasing arterial stiffness in both young and middle-aged adults, however, sitting posture increasing arterial stiffness in young adults. When bout number was increased, the arterial stiffness improvement induced by kneeling posture disappeared in middle-aged adults.

Swiss ball is increasingly popular in the workplace as an alternative to traditional exercise. Our results showed that different arterial stiffness profiles could be elicited by different postures, and increasing exercise dose did not result in more health benefits. People should not have unrealistic expectation that once on-ball balance exercise is performed, health benefit, at least in terms of arterial stiffness, will inevitably occur.

## Data Availability

The original contributions presented in the study are included in the article/supplementary material, further inquiries can be directed to the corresponding authors.

## References

[B1] Beers E. A. Roemmich J. N. Epstein L. H. Horvath P. J. (2008). Increasing passive energy expenditure during clerical work. Eur. J. Appl. Physiol. 103 (3), 353–360. 10.1007/s00421-008-0713-y 18351381

[B2] Buchman-Pearle J. M. Karakolis T. Callaghan J. P. (2022). Does sitting on a stability ball increase fall risk during ergonomic reaching tasks? Appl. Ergon. 102, 103721. 10.1016/j.apergo.2022.103721 35231651

[B3] Calabrese E. J. (2013). Biphasic dose responses in biology, toxicology and medicine: Accounting for their generalizability and quantitative features. Environ. Pollut. 182, 452–460. 10.1016/j.envpol.2013.07.046 23992683

[B4] DeVan A. E. Anton M. M. Cook J. N. Neidre D. B. Cortez-Cooper M. Y. Tanaka H. (2005). Acute effects of resistance exercise on arterial compliance. J. Appl. Physiol. 98 (6), 2287–2291. 10.1152/japplphysiol.00002.2005 15718412

[B5] Dickin D. C. Surowiec R. K. Wang H. (2017). Energy expenditure and muscular activation patterns through active sitting on compliant surfaces. J. Sport Health Sci. 6 (2), 207–212. 10.1016/j.jshs.2015.10.004 30356581 PMC6188989

[B6] Farlie M. K. Robins L. Keating J. L. Molloy E. Haines T. P. (2013). Intensity of challenge to the balance system is not reported in the prescription of balance exercises in randomised trials: A systematic review. J. Physiother. 59 (4), 227–235. 10.1016/s1836-9553(13)70199-1 24287216

[B7] Garber C. E. Blissmer B. Deschenes M. R. Franklin B. A. Lamonte M. J. Lee I. M. (2011). American college of Sports medicine position stand. Quantity and quality of exercise for developing and maintaining cardiorespiratory, musculoskeletal, and neuromotor fitness in apparently healthy adults: Guidance for prescribing exercise. Med. Sci. Sports Exerc 43 (7), 1334–1359. 10.1249/MSS.0b013e318213fefb 21694556

[B8] Haskell W. L. (1994). J.B. Wolffe memorial lecture. Health consequences of physical activity: Understanding and challenges regarding dose-response. Med. Sci. Sports Exerc 26 (6), 649–660. 10.1249/00005768-199406000-00001 8052103

[B9] Haskell W. L. Lee I. M. Pate R. R. Powell K. E. Blair S. N. Franklin B. A. (2007). Physical activity and public health: Updated recommendation for adults from the American college of Sports medicine and the American heart association. Circulation 116 (9), 1081–1093. CIRCULATIONAHA.107.185649 [pii]. 10.1161/CIRCULATIONAHA.107.185649 17671237

[B10] Ikebe H. Cho N. Matsumoto N. Ishido M. Nakamura T. Nishiwaki M. (2022a). Regular exercise ball training reduces arterial stiffness in sedentary middle-aged males. J. Phys. Ther. Sci. 34 (5), 386–392. 10.1589/jpts.34.386 35527848 PMC9057684

[B11] Ikebe H. Takiuchi S. Oi N. Takayanagi Y. Makino A. Itoh M. (2022b). Effects of trunk stretching using an exercise ball on central arterial stiffness and carotid arterial compliance. Eur. J. Appl. Physiol. 122 (5), 1205–1216. 10.1007/s00421-022-04912-8 35220498

[B12] Ishigaki T. Ueta K. Imai R. Morioka S. (2016). EEG frequency analysis of cortical brain activities induced by effect of light touch. Exp. Brain Res. 234 (6), 1429–1440. 10.1007/s00221-015-4545-9 26758719

[B13] Kurayama T. Tadokoro Y. Fujimoto S. Komiya Z. Yoshida S. Chakraborty S. (2013). A comparison of the movement characteristics between the kneeling gait and the normal gait in healthy adults. Gait Posture 37 (3), 402–407. 10.1016/j.gaitpost.2012.08.009 22963826

[B14] Kuusik K. Kepler T. Zilmer M. Eha J. Vahi M. Kals J. (2019). Effects of remote ischaemic preconditioning on arterial stiffness in patients undergoing lower limb angiographic procedures: A randomised clinical trial. Eur. J. Vasc. Endovasc. Surg. 58 (6), 875–882. 10.1016/j.ejvs.2019.06.004 31648881

[B15] Li Y. Bopp M. Botta F. Nussbaumer M. Schäfer J. Roth R. (2015). Lower body vs. Upper body resistance training and arterial stiffness in young men. Int. J. Sports Med. 36 (12), 960–967. 10.1055/s-0035-1549921 26212244

[B16] Liu W. L. Lin Y. Y. Mündel T. Chou C. C. Liao Y. H. (2022). Effects of acute interval exercise on arterial stiffness and cardiovascular autonomic regulatory responses: A narrative review of potential impacts of aging. Front. Cardiovasc Med. 9, 864173. 10.3389/fcvm.2022.864173 35620510 PMC9127236

[B17] Menêses A. L. Forjaz C. L. de Lima P. F. Batista R. M. Monteiro Mde F. Ritti-Dias R. M. (2015). Influence of endurance and resistance exercise order on the postexercise hemodynamic responses in hypertensive women. J. Strength Cond. Res. 29 (3), 612–618. 10.1519/jsc.0000000000000676 25264665

[B18] Mezzarane R. A. Kohn A. F. (2008). Postural control during kneeling. Exp. Brain Res. 187 (3), 395–405. 10.1007/s00221-008-1308-x 18283443

[B19] Miyachi M. (2013). Effects of resistance training on arterial stiffness: A meta-analysis. Br. J. Sports Med. 47 (6), 393–396. 10.1136/bjsports-2012-090488 22267567

[B20] Okamoto T. Masuhara M. Ikuta K. (2013). Low-intensity resistance training after high-intensity resistance training can prevent the increase of central arterial stiffness. Int. J. Sports Med. 34 (5), 385–390. 10.1055/s-0032-1312604 23041961

[B21] Okamoto T. Masuhara M. Ikuta K. (2009). Upper but not lower limb resistance training increases arterial stiffness in humans. Eur. J. Appl. Physiol. 107 (2), 127–134. 10.1007/s00421-009-1110-x 19533164

[B22] Peultier-Celli L. Lion A. Buatois S. Watfa G. Gueguen R. Benetos A. (2021). Relation of arterial stiffness with postural control in older people. Eur. Geriatr. Med. 12 (4), 871–879. 10.1007/s41999-021-00468-6 33687696

[B23] Ramondou P. Hersant J. Bernardeau E. Moumneh T. Feuilloy M. Henni S. (2021). Kneeling-induced calf ischemia: A pilot study in apparently healthy European young subjects. Eur. J. Appl. Physiol. 121 (11), 3031–3040. 10.1007/s00421-021-04764-8 34254181

[B24] Sekendiz B. Cuğ M. Korkusuz F. (2010). Effects of Swiss-ball core strength training on strength, endurance, flexibility, and balance in sedentary women. J. Strength Cond. Res. 24 (11), 3032–3040. 10.1519/JSC.0b013e3181d82e70 20940644

[B25] Snarr R. L. Langford E. L. Ryan G. A. Wilhoite S. (2019). Cardiovascular and metabolic responses of active sitting while performing work-related tasks. Ergonomics 62 (9), 1227–1233. 10.1080/00140139.2019.1633476 31204597

[B26] Synnott A. Dankaerts W. Seghers J. Purtill H. O'Sullivan K. (2017). The effect of a dynamic chair on seated energy expenditure. Ergonomics 60 (10), 1384–1392. 10.1080/00140139.2017.1324114 28449637

[B27] Yoon E. S. Jung S. J. Cheun S. K. Oh Y. S. Kim S. H. Jae S. Y. (2010). Effects of acute resistance exercise on arterial stiffness in young men. Korean Circ. J. 40 (1), 16–22. 10.4070/kcj.2010.40.1.16 20111648 PMC2812793

[B28] Young K. J. Je C. W. Hwa S. T. (2015). Effect of proprioceptive neuromuscular facilitation integration pattern and Swiss ball training on pain and balance in elderly patients with chronic back pain. J. Phys. Ther. Sci. 27 (10), 3237–3240. 10.1589/jpts.27.3237 26644682 PMC4668173

[B29] Yu W. Cha S. Seo S. (2017). The effect of ball exercise on the balance ability of young adults. J. Phys. Ther. Sci. 29 (12), 2087–2089. 10.1589/jpts.29.2087 29643579 PMC5890205

[B30] Zheng L. Zhang X. Zhu W. Chen X. Wu H. Yan S. (2015). Acute effects of moderate-intensity continuous and accumulated exercise on arterial stiffness in healthy young men. Eur. J. Appl. Physiol. 115 (1), 177–185. 10.1007/s00421-014-3008-5 25260247

[B31] Zhou Z. Tao X. Zhang Y. Zhu W. (2022). Acute effects of one-leg standing on arterial stiffness in older women: Role of the vision condition and standing dose. Front. Physiol. 13, 1017486. 10.3389/fphys.2022.1017486 36246140 PMC9565544

